# A Hybrid Search Algorithm for Swarm Robots Searching in an Unknown Environment

**DOI:** 10.1371/journal.pone.0111970

**Published:** 2014-11-11

**Authors:** Shoutao Li, Lina Li, Gordon Lee, Hao Zhang

**Affiliations:** 1 College of Communication Engineering, Jilin University, Changchun, Jilin Province, China; 2 Department of Electrical & Computer Engineering, San Diego State University, San Diego, California, United States of America; 3 Symbol Computation and Knowledge Engineering of Ministry of Education, College of Computer Science and Technology, Jilin University, Changchun, China; Peking University, China

## Abstract

This paper proposes a novel method to improve the efficiency of a swarm of robots searching in an unknown environment. The approach focuses on the process of feeding and individual coordination characteristics inspired by the foraging behavior in nature. A predatory strategy was used for searching; hence, this hybrid approach integrated a random search technique with a dynamic particle swarm optimization (DPSO) search algorithm. If a search robot could not find any target information, it used a random search algorithm for a global search. If the robot found any target information in a region, the DPSO search algorithm was used for a local search. This particle swarm optimization search algorithm is dynamic as all the parameters in the algorithm are refreshed synchronously through a communication mechanism until the robots find the target position, after which, the robots fall back to a random searching mode. Thus, in this searching strategy, the robots alternated between two searching algorithms until the whole area was covered. During the searching process, the robots used a local communication mechanism to share map information and DPSO parameters to reduce the communication burden and overcome hardware limitations. If the search area is very large, search efficiency may be greatly reduced if only one robot searches an entire region given the limited resources available and time constraints. In this research we divided the entire search area into several subregions, selected a target utility function to determine which subregion should be initially searched and thereby reduced the residence time of the target to improve search efficiency.

## Introduction

Robotic urban search and rescue operations are a challenging yet promising research area [1]–[4], which has significant application potential, as has been seen during rescue and recovery operations of disaster events, i.e., the Japan Earthquake in March 2011 [5]. The searching problem is an integral part of many robotic applications ranging from planetary exploration, examination of hazardous environments, rescue operations and warfare, to domestic applications. Robots provide a means to minimize human exposure to harmful situations while providing a mechanism to perform potentially life-saving operations.

The usage of robotic platforms in treacherous environments, in fact, has become a necessity in present day society. There are many researchers investigating this area such as [6]–[9]. Perc and Szolnoki [10] reviewed research in coevolutionary games and also gave a didactic description of potential pitfalls and misconceptions associated with the subject. Hoff et al. [11] suggested a dual agent system requiring two algorithms for searching robots serving as scouts or harvesters during the search. The scouts are designed to be sensor-orientated multiple robots that can perform a more efficient search and collection in a larger area than a singular robot could accomplish. Darvishzadeh in [12] proposes an improved distance-based POS algorithm, which has produced better results than others, but only for a single target; furthermore, the robot requirements for his experiments are relatively high. Sisso et al. in [13] proposes an info-gap approach to the multi-agent search problem under severe uncertainty. The strategy uses a decision making architecture and may be useful in various scenarios; however, it assumes that a database for the search exists. When prior data is known to be very reliable, one might rightfully choose to maximize the expected utility. Caiand Yang [14] put forward an improved particle swarm optimization (PSO) based approach whereby a team of mobile robots cooperate in the search for targets in complex unknown environments. The authors apply improved cooperation rules for a multi-robot system using a potential field function, which acts as the fitness function for the PSO. The main improvements are the district-difference degree and dynamic parameter tuning. Darvishzadeh in [15] presents a framework for a modified PSO algorithm (MPSO) in a multi-robot system for searching tasks in real-world environments. In this paper, we modify this algorithm to optimize the total path traveled by robots. Tang and Eberhardin [16] designed a new approach, Extremum Seeking (ES) which takes into account the mechanical properties that the robot utilizes when conduction a target search. In order to avoid robot localization as well as compensate for noise due to feedback and measurement errors, ES aids the mechanical Particle Swarm Optimization (PSO). As stated by Tang and Eberhard: “The ES based method is capable of driving robots to the purposed states generated by the mechanical PSO without the necessity of robot localization.” This allows the whole robot swarm to approach their target together as a coordinated team. Zhang et al. in [17] investigates the evolution of cooperation among selfish individuals in the stochastic strategy spatial prisoner's dilemma game. The concept of particle swarm optimization was originally introduced within a simple model of social dynamics that can describe the formation of a swarm. Essentially, particle swarm optimization foresees changes in the velocity profile of each player, such that the best locations are targeted and eventually occupied.

In disaster-related situations, finding the targets (humans) is the most important task. Based on the research methods just discussed in the introductory literature survey, we investigated a new hybrid search algorithm inspired by the foraging behavior of creatures searching for food in the natural world, and focused on the process of feeding and defining individual coordination characteristics. Our new hybrid algorithm was tested using by a swarm of robots in an unknown environment and has proved capable of speeding up the search process for targets that are both within and outside a sensible region. This hybrid algorithm also guarantees finding all the targets. Compared to existing work in this area, our approach reduces the complexity, which in turn makes it easier to implement and more efficient. This hybrid search algorithm can be applied to a variety of places such as searching for survivors in case of fire or mine disaster, and it could even be used for outer space exploration. In addition, our proposed target utility function requires less resource from the robots for computation tasks, which makes our algorithm applicable to a wide range of robots in practice.

The rest of this paper is organized as follows: In the Materials and Methods section, we present our hybrid search algorithm based on both random search and the DPSO search. We then define our robotic control structure, communication mechanism, and map storage strategy. In the Results and Discussion section, we propose a target utility function and introduce the simulation platform followed by experimental results. The Conclusion summarizes the benefits of our new hybrid search algorithm compared with other algorithms and recommends future research.

## Materials and Methods

### The hybrid search algorithm based on a random search algorithm and DPSO search algorithm

While investigating how zoologists model predatory behavior of animals, we found that in spite of the different animal species with vastly different body structures, their predatory behavior is surprisingly similar. When animals prey, in the absence of a food source or when prey signs are found indicating possible food sources, the predators search the entire space following a certain direction. Once a sign of a prey is found, they slow down their pace and concentrate on a smaller region for a more intensified regional search. If, after a period of time, the prey is not found, a predator will abandon the concentrated area, and continue searching the remaining open space.

**Figure 1:**
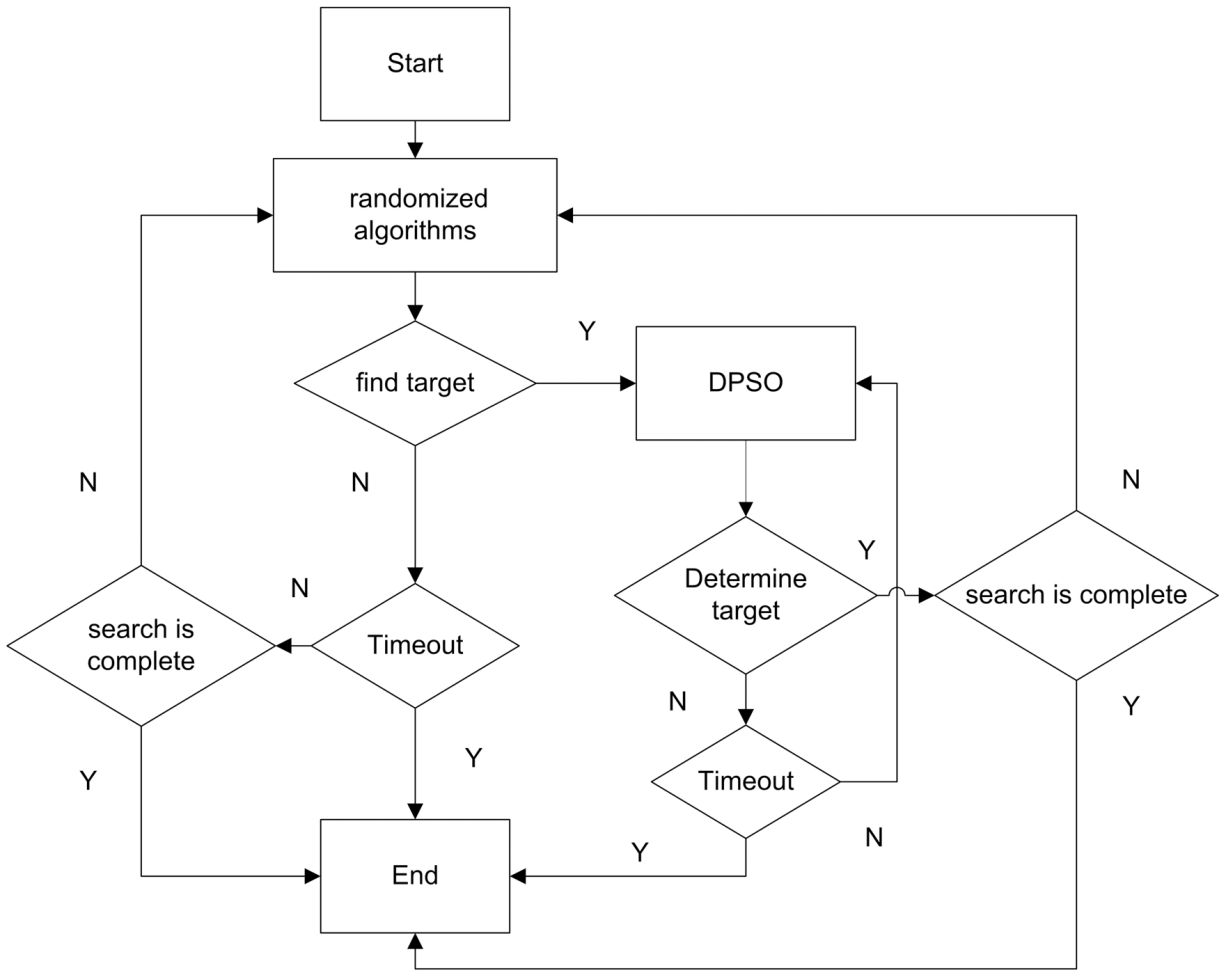
Hybrid search algorithm flow chart.

Inspired by this predation strategy, we proposed a hybrid search algorithm, based on a random search algorithm and DPSO search algorithm. From the beginning of the search until the target is identified, the robot's velocity is a constant. If a robot can't find a target or targets, it will use a random search algorithm for a global search. When a robot finds a target or targets, it will stop using the random search algorithm and start using the DPSO search algorithm, employing the concentration value of the target to change its velocity, and at the same time, it will work with other robots within the communication range to determine the target's position. A hybrid search algorithm flow chart is shown in Figure 1.

In this paper, the velocity and position update of the random search algorithm are defined as follows:
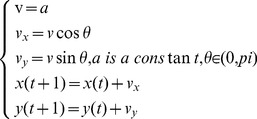
(1)


Here *v(t)* represents the velocity of a robot, *θ* represents the angle between the velocity vector and the *x*-axis; and Δ*t* is the time interval. In (1), we have selected Δ*t* = 1.

The velocity and position update of DPSO is shown in (2). Here, the initial value of the velocity, *v*(*t*), is set to *a*, and then changes with time. Further, *θ∈ (0, π)* because Δ*t* = 1. Also, 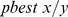 represents the best position of a robot alone the 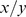-axis in the search process, and 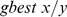 represents the best position of all robots within the communication range along the 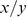-axis in the search process. Finally, *ω*, *c_1_* and *c_2_* are time-varying weights, tuned according to the particular scenarios, where 0<ω<2, 0<c_1_<1, and 0<c_2_<1.
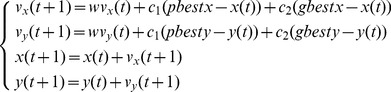
(2)


The DPSO algorithm is called dynamic for two reasons: 1) the numbers of the population, 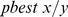 and 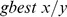 are determined by the communication mechanism which establishes a priority, and 2) the random search algorithm and DPSO search algorithm continuously transition during the search process.

In (3), we define a signal concentration function as the DPSO fitness function. The maximum measurable distance of the target signal is D. Targets can be measured when the distance is less than or equal to D. Furthermore, Q is a constant of the target energy, d_i_ is the distance between robot i and the target, and 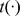 is a function of growth, which increases with time as shown in (4).
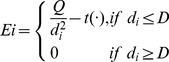
(3)

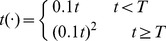
(4)


**Figure 2:**
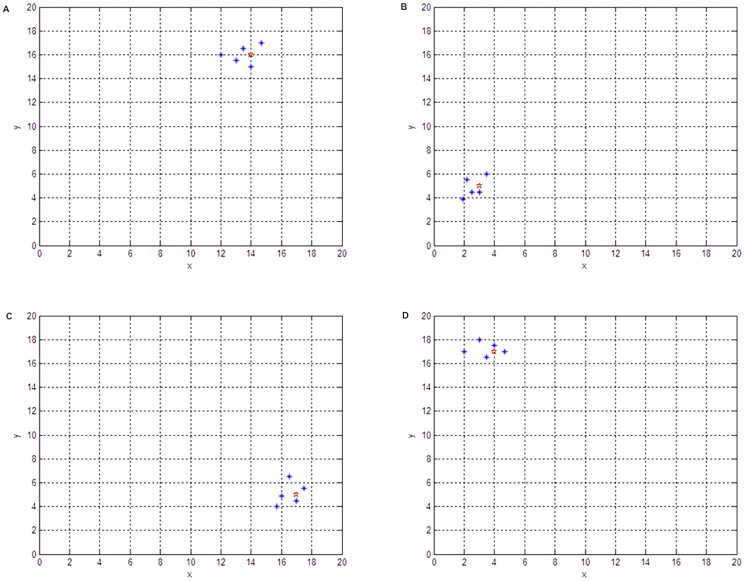
Algorithm Verification (one single target point existing in the different searching subregion). The red pentacle indicates the position of the target set beforehand that needs to be found by the robots in the subregion. The blue asterisks represent the robots. (A) Target being set in the upper right side; (B) Target being set in the lower left side; (C) Target being set in the lower right side; (D) Target being set in the upper left side.

In order to verify the effect of the hybrid search algorithm proposed here, we used particles to simulate a search. The simulation results are shown in Figure 2 and discussed later in Section 4. The initial state of every search is same: There are five particles to search: The blue dots are searching particles; the red dots represent the target. From Figure 2 we see that no matter where the target is and no matter what the distance is of particles from the target, a number of particles will always reside around the target, and the rest of the particles will be nearby. Hence, one can easily carry out the next step of processing, such as transportation, and so on.

**Figure 3:**
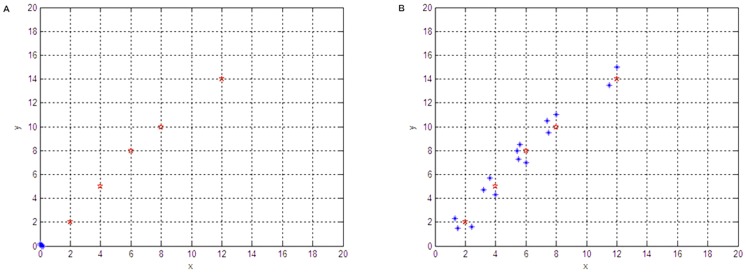
Algorithm Verification (multiple targets existing in the searching subregion). The red pentacles indicate the position of the target set beforehand that need to be found by the robots in the subregion. The blue asterisks represent the robots. (A) Original state of the robots; (B) Search Results of the Robots.

When there are multiple targets, our proposed algorithm is equally effective. The simulation results shown in Figure 3(A) represent the initial state of each particle. The blue dots represent searching particles, and the red dots represent the target point. From Figure 3(B), one can see that each target has been found by several particles.

### Robot control structure

**Figure 4:**
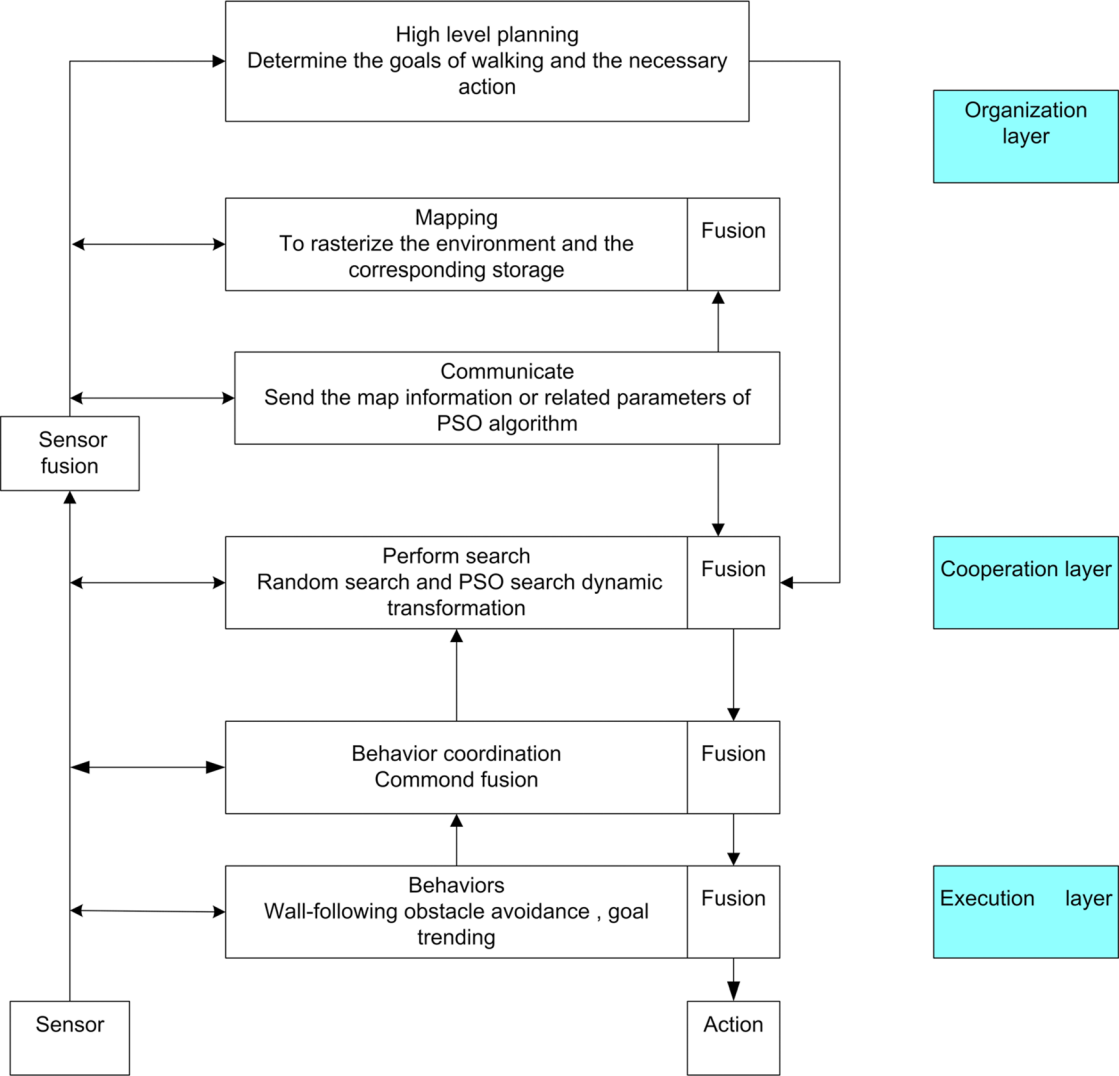
Robot control structure.

The robot control structure is shown in Figure 4. The control structure is divided into three levels: organization layer, cooperation layer and execution layer. The organization layer determines the goals for moving (walking or rolling) and the necessary actions for mobility while information fusion and cooperation during the search process are in the cooperation layer. Finally, in the execution layer, the robots perform operations such as wall-following, obstacle avoidance, and goal trending. Here the organization layer and cooperation layer are very important as they determine whether the search can be accomplished efficiently.

**Figure 5:**
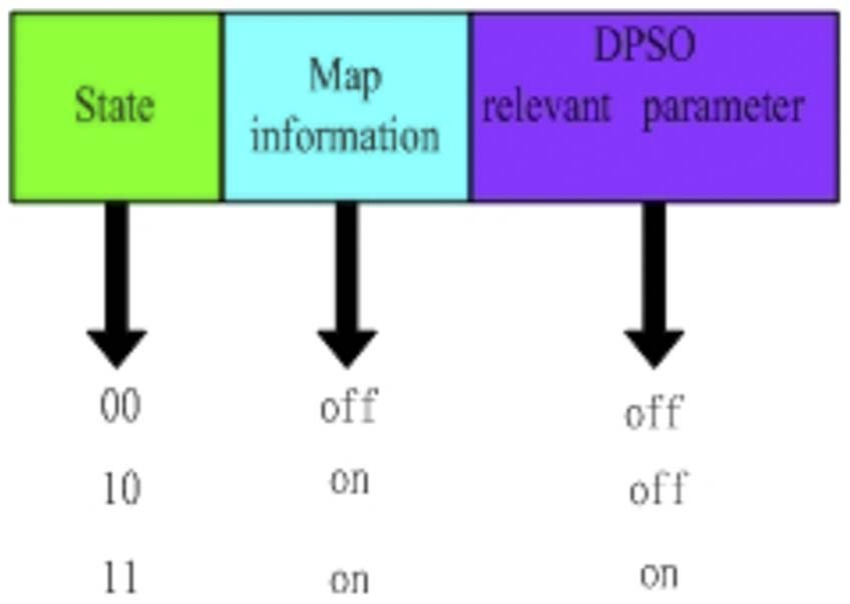
Communication Packet Structure.

In this paper, robots use local communication for delivering information. This can effectively reduce the burden of communication and is easier to apply. The robot communication packet structure is shown in Figure 5. Communication consists of three parts: 1) the *state* which denotes the communication method (note that because map information is always delivered, there is not a state 01); 2) *map information* (This information includes data about the grid associated with the subregions and whether or not a subregion has been searched); and 3) *related-DPSO parameters,* which include the parameters for the DPSO algorithm.

During the entire search process, the robot exhibits three types of behavior: (1) a random search behavior, i.e., a robot in the search space uses the random search algorithm during the initial search; this type of behavior was denoted as *liberty*; (2) an organizational behavior, i.e., when a robot detects a target, it will inform other robots within its communication range to help determine the target position, and it will inform others about its position (denoted as *organizer*), and (3) a cooperative behavior where another robot helps the organizer find the targets (denoted as *collaborator*). These three types of robot behavior act continuously during the search procedure.

We can analyze robot behavior conversion during the search process. For example, suppose robots are designated by R1, R2, R3, R4, and R5 as shown in Figure 2. We used *0* to represent *liberty*, *1* to represent *organizer*, *2* to represent *collaborator*. In this example, the target is located at the top of Figure 2; so, at the beginning of the search, all robots are in state *0*. We then select a few key points in time during the search process to analyze the state of each robot. *T1* is the time in which a robot discovers a target. *T2* is the time after the communication and *T3* refers to the state when every robot is in the search process at some moment. *T4* refers to the state after the robots find the target position. From Table1, we can see that, at T1, R1 found a target, after which its state changed from *0* to *1*, while the other robots' state remains at *0* at T2 since they did not discover the target. Since, in this example, R2, R3, R4, and R5 are all in the communication range of R1, their states change to *2*. As the search progresses, the concentration value of the target signal of R2 is larger than R1; so the state for R2 change from*2* to *1*; the state of R1 switches from *1* to *2* at T3, and, at T4, after the robots determine the position of the target, and all states of the robots convert to *0*.

**Table 1:** The State of Each Robot.

	T1	T2	T3	T4
R1	0→1	1	1→2	2→0
R2	0	0→2	2→1	1→0
R3	0	0→2	2	2→0
R4	0	0→2	2	2→0
R5	0	0→2	2	2→0

In Table 1, “T” refers to Time, “R” refers to Robot and the Arabic numerals refer to the state of each robot.

Each robot has two types of memory: one is permanent memory, which records the status of each subregion search and one is erasable memory. If the current subregional search is completed, the robot's state is recorded as *1*; otherwise, it is *0*. The robot's erasable memory records the state of subregional grids. If one is searched, the subgrid's state changes to *1*; otherwise, it is *0*. When the subregional search is completed, we inform memory A, which automatically clears the memory. We can then go to the next subregion to start recording again, which greatly reduces the storage burden.

## Results and Discussion

This section presents some results of applying the hybrid search algorithm to a set of robots searching a region. The scenarios include the cases of no targets, one target, one target and multiple targets and a comparison is also performed between the proposed hybrid method developed here and a pure random search.

### A target utility function

Because of the large search area, if one searches the entire region all at once, the search efficiency may be greatly reduced, given the limited resources available and time constraints. In this paper, in order to improve search efficiency, we used a grid method for environmental modeling. That is, the entire search area was divided into several subregions. Employing the Principle of Priority Discovery for targets, to reduce the residence time of the target, consider a target utility function (5), to select which subregion should be initially searched. 
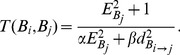
(5)


In (5) 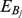 denotes the concentration of the target signal detected in subregion 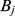, and 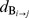 is the distance between the center of subregion 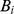 and 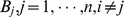. The coefficients 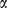 and 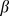 are weights. When 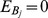, there is no target within the subregion. Note that 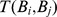 means that the target utility value of subregion 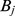 can be detected within subregion 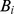. Each robot has its own identification tag, and the first region to be searched is selected by the first robot (tagged as number 1); then, the choice of the second subregion to be searched is determined by the first robot to complete the searching of the first subregion because the concentration of signals in each region is random.

**Figure 6:**
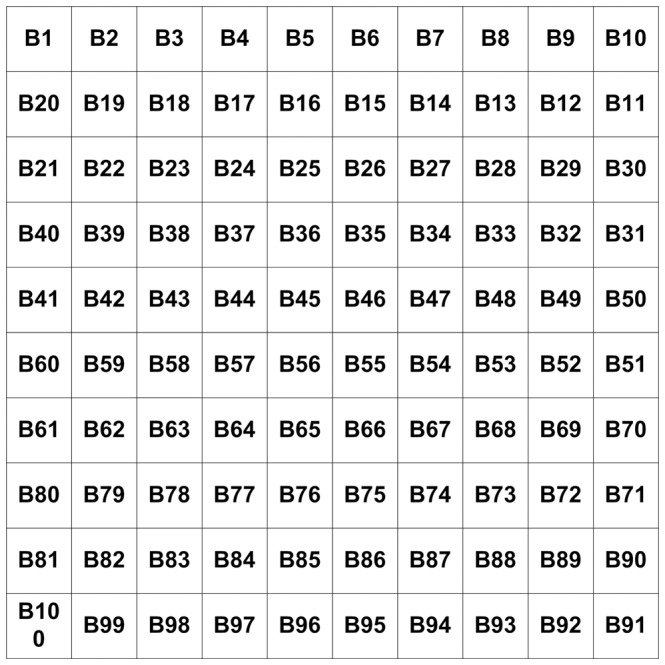
Region Division.

**Figure 7:**
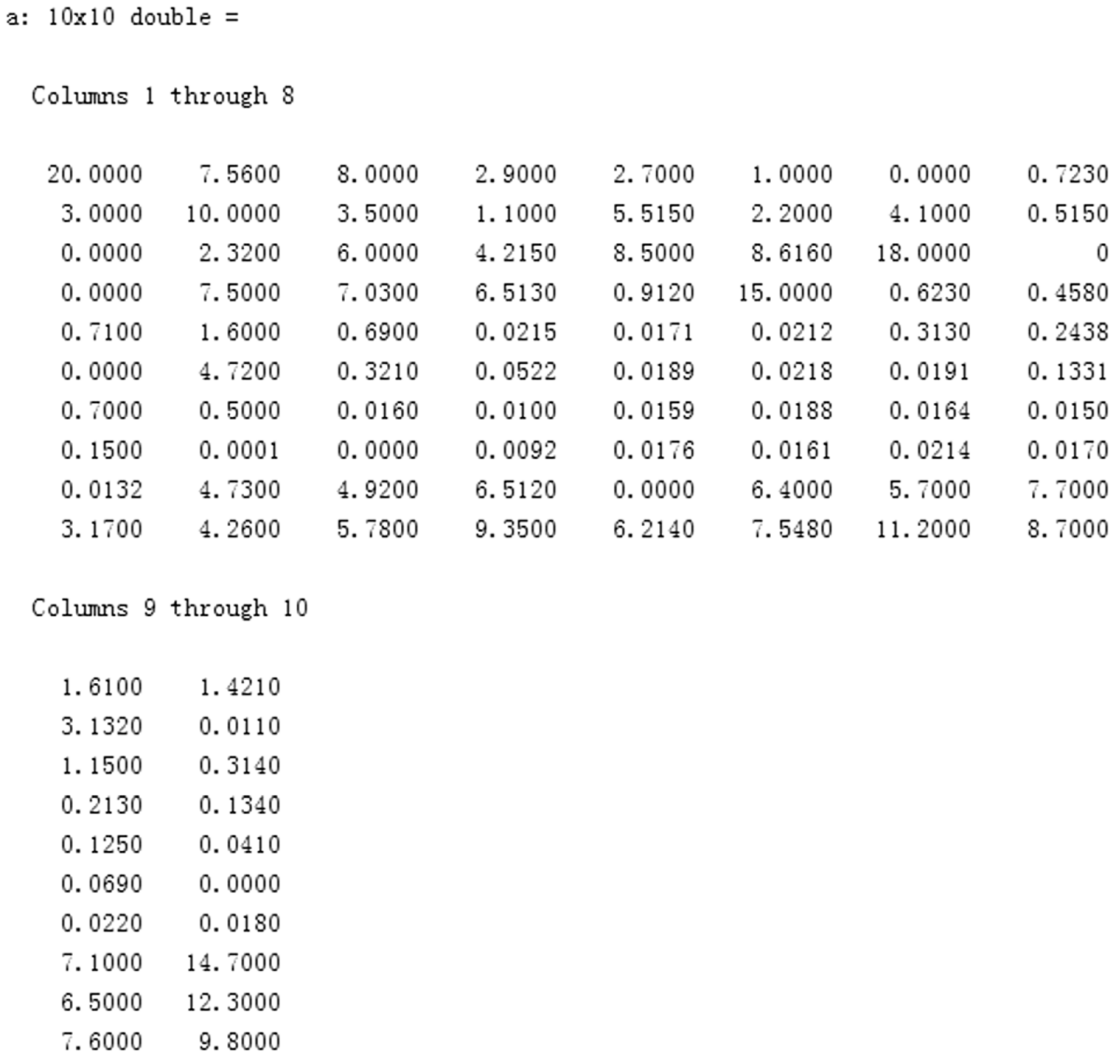
Values of subregions.

In order to prove the effect of the target utility function developed in this paper, suppose all of region is divided into 100 subregions (shown in Figure 6). Assume that the concentration values of these subregions can be detected, as shown in Figure 7. This figure shows the concentration values of these subregions.

**Figure 8:**
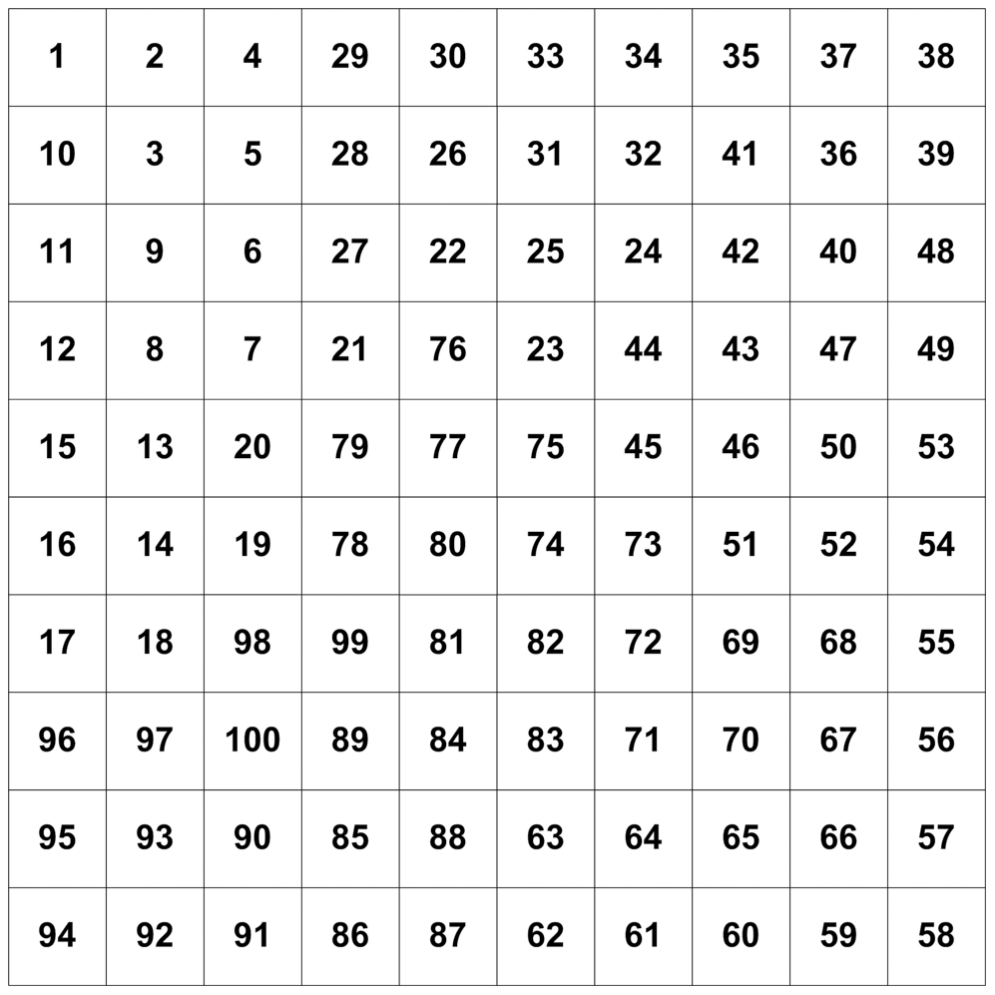
Search route (The number of the subregions indicate the sequence of the robot search route).

Suppose the center-to-center distance of two adjacent subregions is10; further let the angle region distance be 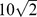, and select 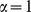 and 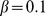. From (3), subregion B1 has the highest target utility value; so we first select this subregion to search. When the searching of subregion B1 is completed, we use (3) again to select the next subregion to search, which is subregion B2 in this example. This procedure is repeated resulting in the search route shown in Figure 8.

As discussed above, we can see that when using the utility function to select which subregion should be searched, the search becomes more flexible, and the strong signal concentration of subregions in the region can be searched as soon as possible.

To search in a fixed manner, such as from top to bottom or from left to right mandates a search pattern that will ignore a noticeably strong regional concentration of signals to run its pattern. This was the case found in region B19. Such an inflexible method finds the target too late to be usable for processing, which wastes time and is very inefficient for any research effort.

### Simulation results

**Figure 9:**
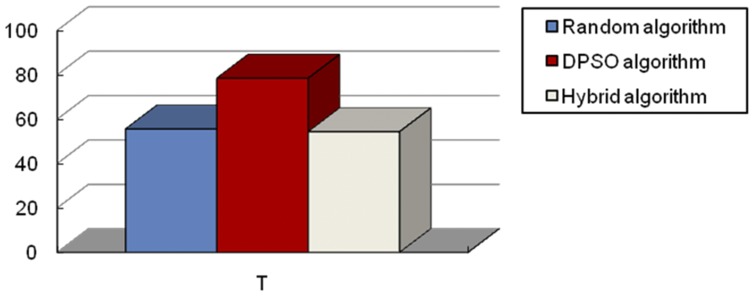
Comparison results of three algorithms with no target existing in the subregion.

In order to validate the performance of the proposed method, we compared the experimental simulation results by using a single search algorithm with that of hybrid search algorithm in different subregions, which are shown in Figure 9 to Figure12. Before we discuss the results, let us define some of the evaluation criteria first.

T: The total searching time of the whole region.

Tn: The searching time of the robots detecting the n-th target by cooperation, where n is a natural number, i.e., n = 1, 2, 3…

N: The total number of targets that the robots can detect by cooperation in the whole searching region, where N is a natural number, i.e., N = 1,2,3…

Experiment I: No target exists in the searching subregion.

We selected a searching region where no target existed in each subregion, and compared the searching time of the robots using a single algorithm (i.e., either a random algorithm or a DPSO algorithm) and the hybrid algorithm. Figure 9 shows the simulation results which compare results of the total searching time (T). We can see that the results from the random algorithm and the hybrid algorithm do not differ much from each other because when there is no target existing in the subregion, the hybrid algorithm becomes only a random searching one. On the other hand, the DPSO algorithm spends a little more time searching because the DPSO algorithm itself is much more complex than the random algorithm and costs more in time during the whole searching process.

From the simulation results above, we conclude that in a region with no targets, the three algorithms have similar performance. The next experiment used other experimental environments to test the performance of the three algorithms.

Experiment II: Only a single target exists in the searching subregion.

**Figure 10:**
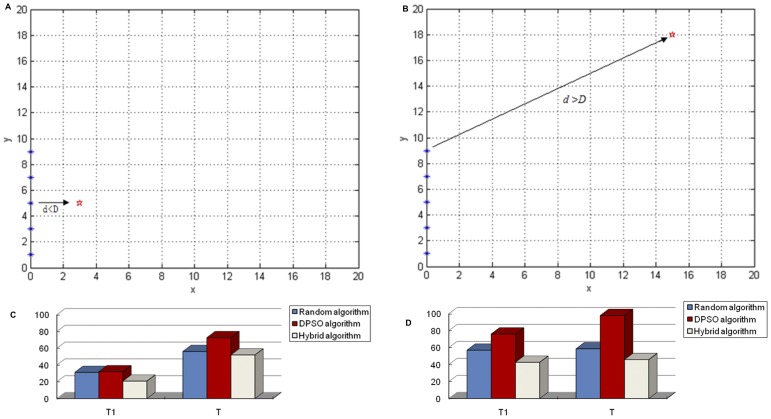
The initial position of the robots and comparison results of three algorithms with a single target in the subregion. D: The distance of the robot's perceiving ability. d: The distance between the start point to the nearest robot. T: The total searching time of the whole region. T1: The searching time of the robots detecting the first target by cooperation.(A).d<D; (B).d>>D; (C). The comparison results of three algorithms where d<D; (D). The comparison results of three algorithms where d>>D.

Here, we chose an environment with only one single target in the subregion to be searched. In this case, we changed the location of the target twice. First we set the location of the target to be far away from the starting point of the robots (shown in Figure 10(A)), and second the distance is longer than the robot's perception ability to detect the targets (shown in Figure 10(B)). The simulation result is shown in Figure 10(A). Next we set the location of the target to be close to the starting point of the robots and the distance was within the robot's perception ability. The result is shown in Figure 10(C). In these two experiments, we chose two evaluation criteria to test the performance, one being the time used to find the first target (T1), and the other being the total searching time (T). Because the robots don't know how many targets we have set in the whole region, they have to finish the whole searching process. After using the single algorithms and the hybrid algorithm separately, we collected the experiment results shown in Figure 10 (C) and (D).

We can see, from Figure 10 (A), that the time to determine the first target (T1) by using the hybrid algorithm is shorter than that of the DPSO algorithm and the random algorithm when the target is outside the range of the robots' perception. Next, from Figure 10(B), we can see that time T1 of the hybrid algorithm and the DPSO algorithm are almost the same when the target is within the perception range of the robots. In addition, the time T1 of these two algorithms is better than the random algorithm in this case. One question to consider is: Why did we get different performance results in almost the same environment when comparing the two algorithms to the random one? The reason is that the location of the target was different in the two experimental environments. In one case the target was very far away from the starting point of the robots and in the other case, the target is near to the starting point. In Figure 10(B) the distance between the target and the starting point is within the robot's sensing ability. Therefore, the hybrid algorithm switches from the random algorithm immediately to the DPSO algorithm after searching for a short period of time; hence, the robots almost always use the DPSO algorithm all the way to the target, but they will use the random algorithm transitorily. This is why these two algorithms spend almost the same amount of time in determining the target. Now, in the other criterion, the total searching time (T), we can see that the hybrid algorithm had the best results, and the DPSO algorithm spent the most time in searching.

From the simulation experiment above, we can draw several conclusions. First, in the environment with only one target, the hybrid algorithm had the best performance whether the target was close to the starting point of the robots or not. Second, the determination time of both the DPSO and the hybrid algorithm were almost identical when the distance from the target to the starting point was short, and the determination time of the hybrid algorithm was shorter than DPSO when the distance was longer than the robots' perception ability.

Experiment III: Multiple targets exist in the searching subregion.

**Figure 11:**
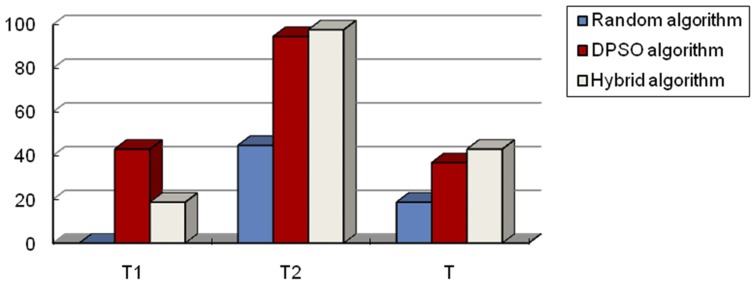
The comparison results of three algorithms with two targets existing in subregion. T: The total searching time of the whole region. Tn: The searching time of the robots detecting the n-th target by cooperation, where n is a natural number, i.e., n = 1, 2, 3… here n = 2.

**Figure 12:**
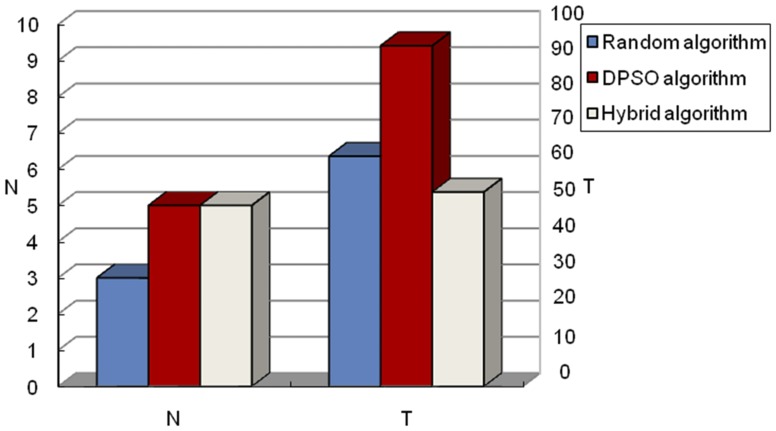
The comparison results of three algorithms with five targets existing in subregion. T: The total searching time of the whole region. N: The total number of targets that the robots can detect by cooperation in the whole searching region, where N is a natural number, i.e., N = 1, 2, 3….The left ordinate axis is the number of targets robots found, and the right ordinate axis presents algorithm search time. This figure shows that the random algorithm spent shorter time in searching, but the number of targets found was less than that found by the other two methods. On the other hand, the proposed hybrid algorithm spent almost the same amount of time in the complete search of the entire subregion, and found all targets; hence the hybrid algorithm is superior to the other two algorithms.

Here we set up two experiments: one having two targets and the other having five targets in a subregion. The simulation results are shown in Figure 11 and Figure 12. T1 represents the time when the first target is determined, and T2 represents the time when the second target is determined. From the results of these two simulations, we can see that the advantages of the hybrid algorithm are more obvious in finding all the targets. First, the hybrid algorithm, unlike the random algorithm, can avoid the problem of missing targets. This can be seen from Figure 11. Time T1 of the random algorithm is 0 because this algorithm cannot find the target. In addition, from Figure 12, the number of targets determined by the random algorithm is less than that of the other two methods. This is because the speed of the robots using the random algorithm remained the same no matter whether they found the target or not. Robots do not change their searching speed according to the distance between them and the target; however, the hybrid algorithm does. This characteristic of the hybrid algorithm to change its speed dynamically according to the distance between robots and targets speeds up the process of searching and identifying targets. The robot will slow down so as not to miss the target upon approach. On the other hand, when it is far away from the target, the robot will increase its speed in order to approach the target as soon as possible. All these hybrid algorithm advantages decrease searching time and improve efficiency. We can see from Figure 12 that, although the DPSO algorithm does not miss the targets, it spends more time than that of the hybrid algorithm. This is because, when robots are far away from the target and cannot sense any target, DPSO, which is a single algorithm, cannot switch to the random algorithm like the hybrid algorithm does to speed up the searching process. It should be noted that multi-target searching is more complicated than single target searching. Therefore, the searching time of multi-target is much longer than that of a single one.

From the different groups of simulation results above, our first conclusion states that the proposed hybrid algorithm is superior to the DPSO algorithm and similar to the random algorithm in terms of total searching time. The reason why the hybrid algorithm is similar to the random algorithm is because when multiple targets are existing in the subregion, robots will switch to the DPSO algorithm many times. We know DPSO is more complex than the random algorithm, which leads to the result that the presented hybrid algorithm takes more time to finish the whole multi-targets exiting region than a pure random algorithm even though it can change robots' searching speed. However, more importantly, the hybrid algorithm can guarantee the robots will detect all the targets without losing one, while a randomized algorithm cannot. Concerning the performance of target determination time, the hybrid algorithm performed better than DPSO in almost all cases. The only case where the hybrid algorithm performed similar to DPSO is when the distance between target and the starting point was close. Finally, the hybrid algorithm we propose can guarantee finding all the targets in the whole region.

Based on these conclusions, we see that the proposed Hybrid algorithm can complete the search task without missing a target while ensuring an excellent performance throughout.

## Conclusions

Throughout the simulation, when a random search method was combined with the DPSO method, the search process for targets in unknown environments was improved. Furthermore, using our local communication strategy and our storage strategy greatly reduced the burden on hardware; so the search tasks were easier to complete. Of course, in the simulation studies, we used simulated robots. An area of future research will focus on applying the proposed hybrid method to real robots as well as investigating possible methods to efficiently implement the approach on embedded controllers.
